# Kallikrein gene downregulation in breast cancer

**DOI:** 10.1038/sj.bjc.6601451

**Published:** 2004-01-06

**Authors:** G M Yousef, G M Yacoub, M-E Polymeris, C Popalis, A Soosaipillai, E P Diamandis

**Affiliations:** 1 Department of Pathology and Laboratory Medicine, Mount Sinai Hospital, Toronto, Ontario, Canada; 2 Department of Laboratory Medicine and Pathobiology, University of Toronto, Toronto, Ontario, Canada; 3 University of Virginia School of Medicine, Roanoke-Salem Internal Medicine Program, Roanoke, VA 24033, USA

## Abstract

Recent evidence suggests that many members of the human kallikrein gene family are differentially regulated in breast cancer and other endocrine-related malignancies. In this study, we utilised the serial analysis of gene expression (SAGE) and expressed sequence tag (EST) databases of the Cancer Genome Anatomy Project (CGAP) to perform *in silico* analyses of the expression pattern of the 15 human kallikrein genes in normal and cancerous breast tissues and cell lines using different analytical tools such as Virtual Northern blotting, Digital Differential Display and X-profiler. Our results indicate that at least four kallikrein genes (*KLK5, 6, 8*, 10) are downregulated in breast cancer. Probing eight normal and 24 breast cancer SAGE libraries with gene-specific tags for each of the above kallikreins indicated moderate-to-high expression densities in normal breast (27–319 tags per million; tpm, in two to five out of eight libraries), compared to no or low expression (0 – 34 tpm in zero to two libraries out of 24) in breast cancer. These data were verified by screening the EST databases, where all mRNA clones isolated for these genes, except for one in each, were from normal breast libraries, with no clones detected from breast cancer tissues or cell lines (with the exception of *KLK8*). X-profiler comparison of two pools of normal and breast cancer libraries further verified the presence of significant downregulation of expression levels of 4 of the kallikreins genes (*KLK5, 6, 10, 12*). We experimentally verified the downregulation of these four kallikreins (*KLK5*, *6*, *8*, *10* and *12*) by RT – PCR analysis.

Cancer is fundamentally a disease of the genome. A key component in understanding the nature of cancer is the identification of those genes that are directly involved in or are associated with malignancy. In the past two decades, much progress has been made in identifying genes that play direct roles in the development of cancer. The Cancer Genome Anatomy Project (CGAP) aims to catalog all genes expressed during cancer development. Knowledge of these genes can then be utilised to understand the pathogenesis of cancer, to define its molecular signatures and to develop biomarkers for early detection and targets for intervention (Strausberg, 2001).

The Cancer Genome Anatomy Project focused on a strategy for gene identification based on isolation of mRNAs from tissues and generation of libraries from these transcripts. This library of genes, expressed in normal and cancer cells, has been developed through the application of two approaches; the expressed sequence tag (EST) (Adams *et al*, 1993) and the serial analysis of gene expression (SAGE) (Velculescu *et al*, 1995). The SAGE method is similar to the EST approach, in that it provides a sequence tag for a portion of a cDNA. In this approach, the tagged sequences are generally quite short (10–14 bp) and the individual tags are annealed to generate DNA molecules carrying many individual tags. Therefore, each DNA sequencing read generates tags for multiple transcripts. This method has been shown to quantitatively assess transcript levels and is more efficient than traditional EST sequencing. Thus, the opportunity to identify rarely expressed transcripts is increased in the SAGE approach.

Kallikreins are a family of 15 genes clustered together on chromosome 19 (Yousef and Diamandis, 2001,^2003^) and encode for serine protease enzymes (Clements *et al*, 2001; Yousef and Diamandis, 2002b). Prostate-specific antigen (PSA, hK3), a member of this family, is an established tumour marker for prostate cancer (Barry, 2001). Prostate-specific antigen SA was recently found to be expressed in the female breast and might have a prognostic value in breast cancer (Black and Diamandis, 2000). Furthermore, other kallikreins were also found to have prognostic value in breast and other endocrine malignancies (Liu *et al*, 1996; Tanimoto *et al*, 1999; Yousef *et al*, 2000a; Benson *et al*, 2002; Diamandis and Yousef, 2002; Yousef and Diamandis, 2002a,^2003^; Diamandis *et al*, 2003; Luo *et al*, 2003).

In this study, we used an *in silico* analysis approach to examine kallikrein gene expression in normal and cancerous breast tissues and cell lines. We provide evidence that at least four kallikreins are downregulated in breast cancer, and experimentally verified the downregulation of of these kallikreins (*KLK5* and *KLK12*) by RT – PCR.

## MATERIALS AND METHODS

### Serial Analysis of gene expression (SAGE)

All publicly available SAGE data until August 2003 were used for analysis of kallikrein gene expression. We obtained a reliable mapping of UniGene (http://www.ncbi.nlm.nih.gov/Un iGene/) groups to both *Nla*III and *Sau*3A tags from the Serial Analysis of Gene Expression Tag to Gene Mapping (SAGEmap) search tool available through the NCBI web site (http://www.ncbi.nlm.nih.gov/). Each UniGene group consists of all GenBank sequences representing the same human gene. Hereafter, each such group is referred to as a ‘gene’. Tags mapping to more than one kallikrein were excluded.

### Virtual Northern blots (VNB)

The mRNA sequences of the 15 human kallikrein genes were used to identify unique sequence tags of UniGene clusters for each kallikrein (the GenBank reference sequences were used). Two restriction digestion enzymes were used (*Nla*III, *Sau*3A). These sequence tags were then used to determine the levels of expression of different kallikreins in eight normal (six tissues and two cell lines) and 24 breast cancer (12 tissues and 12 cell lines) libraries. Detailed information of these libraries is available from the website of CGAP (http://www.ncbi.nlm.nih.gov/nc icgap/). Analyses were carried out by comparing the proportion of libraries of each type (normal *vs* cancer) that show the expression of each tag in addition to the average expression densities in these libraries. mRNA sequences from the Human Genome Project were used as reference sequences. If more than one tag of the same gene appears in the same library, we only included the one with the highest expression (maximum tpm); the other tag was excluded to avoid inaccurate estimation of expression. Expression levels are displayed as blots with different densities and corrected as tpm to facilitate comparison. Table 1

**Table 1:** Gene-specific SAGE tags used to probe different libraries of the CGAP databases

**Kallikrein**	**Restriction enzyme**	**SAGE tags**	**Unigene cluster**
*KLK3*	*Nla*III	GGATGGGGAT	Hs.171995
		GTGACACAGC	
	*Sau*3A	CACACTGAGA	Hs.171995
		ACGCTTTTGT	
*KLK5*	*Nla*III	TCTCCTGGAC	Hs.50915
	*Sau*3A	CAGGAAACCA	Hs.50915
*KLK6*	*Nla*III	CACTCAATAA	Hs.79361
	*Sau*3A	CAAAAAACCA	Hs.79361
*KLK8*	*Nla*III	GTCTGTGCAG	Hs.104570
	*Sau*3A	TCCCTTAATA	Hs.104570
*KLK10*	*Nla*III	TAAGGCTTAA	Hs.69423
	*Sau*3A	CAGATGCCCA	Hs.69423
*KLK11*	*Nla*III	GTGTGTGCCA	Hs.57771
	*Sau*3A	CAGGAGACGA	Hs.57771
*KLK12*	*Nla*III	AGGAACAACT	Hs.159679
	*Sau*3A	ATGAGGAACA	Hs.159679

Only kallikreins that showed differential expression are shown in this table.

shows the tags and UniGene clusters used for each gene to probe the GCAP libraries.

### Expressed sequence tag (EST) analysis

The full-length mRNA sequence of each kallikrein was compared against the human EST databases of the NCBI. At the time of the study, these databases included seven normal and 14 breast cancer libraries. Expression was calculated for each kallikrein as the number of positive libraries out of the total in each tissue type, in addition to the total number of clones detected in each type.

### X-profiler analysis

X-profiler analyses of kallikrein gene expression were performed by comparing normal and breast cancer libraries available in the SAGE databases. As expression levels of various kallikreins might be different from one cell line to another and in different types of breast cancer, we compared a pool of eight normal breast libraries (from tissues and cell lines) against 24 breast cancer libraries from tissues and cell lines. The X-profiler cut-off value was set at two-fold difference.

### Digital differential display (DDD) analysis

The DDD search engine (Strausberg *et al*, 2001) was used to compare EST expression in normal and cancer libraries. The databases at the time of analysis included seven normal and 14 breast cancer libraries. Libraries with less than 25 clones were excluded from the analysis.

### Normal and malignant breast tissues

Normal breast tissues were obtained from four women undergoing reduction mammoplasties. Breast tumour tissues were obtained from 14 female patients at participating hospitals of the Ontario Provincial Steroid Hormone Receptor Program. The normal and tumour tissues were immediately frozen in liquid nitrogen after surgical resection and stored in this manner until extracted. The tissues were pulverised with a hammer under liquid nitrogen and RNA was extracted as described below. Our protocols were approved by the Institutional Review Board of the University of Toronto.

### Reverse transcriptase polymerase chain reaction (RT – PCR)

Total RNA was extracted from the cell lines or tissues using Trizol reagent (Gibco BRL) following the manufacturer's instructions. RNA concentration was determined spectrophotometrically. Total RNA (2 *μ*g) was reverse-transcribed into first-strand cDNA using the Superscript™ preamplification system (Gibco, BRL, Gaithersburg, MD). The final volume was 20 *μ*l. Two gene-specific primers were designed for each kallikrein (Table 2

**Table 2:** Primers and conditions used for PCR analysis

**Gene**	**Sequence^a^**	**Product length^b^**	***T*_a_^c^**	**Number of cycles**
*KLK5*	GTCACCAGTTTATGAATCTGGGC	328	60	35
	GGCGCAGAACATGGTGTCATC			
*KLK6*	GAAGCTGATGGTGGTGCTGAGTCTG	454	60	35
	GTCAGGGAAATCACCATCTGCTGTC			
*KLK8*	GCCTTGTTCCAGGGCCAGC	416	65	35
	GCATCCTCACACTTCTTCTGGG			
*KLK10*	GGAAACAAGCCACTGTGGGC	468	60	35
	GAGGATGCCTTGGAGGGTCTC			
*KLK12*	GAGCAGATCCGGCACAGCGG	425	69	35
	TCTTGTCCACAGGGCCCCACAG			
	GGCAGGGCGCAGCGCTCC			
*KLK15*	CTACGGACCACGTCTCGGGTC	459	65	35
	GACACCAGGCTTGGTGGTGTTG			
*β-actin*	ATCTGGCACCACACCTTCTA	835	62	35
	CGTCATACTCCTGCTTGCTG			

a All sequences are mentioned from the 5′ to 3′ direction.

b In base pairs.

c Annealing temperature.

), and PCR was carried out in a reaction mixture containing 1 *μ*l of cDNA, 10 mM Tris-HCl (pH 8.3), 50 mM KCl, 1.5 mM MgCl_2_, 200 *μ*M dNTPs (deoxynucleoside triphosphates), 150 ng of primers and 2.5 U of AmpliTaq Gold DNA polymerase (Roche Molecular Systems, Branchburg, NJ, USA) on a Perkin-Elmer 9600 thermal cycler. The cycling conditions were 94°C for 15 min followed by 40 cycles of 94°C for 30 s, annealing for 1 min (Table 2) and a final extension step at 63°C for 10 min. Equal amounts of PCR products were electrophoresed on 2% agarose gels and visualised by ethidium bromide staining. All primers for RT – PCR spanned at least two exons to avoid contamination by genomic DNA. Expression of the house-keeping gene (actin) was examined at the same experimental conditions for each kallikrein, but the gel was run separately.

To verify the identity of the PCR products, they were cloned into the pCR 2.1-TOPO vector (Invitrogen, Carlsbad, CA, USA) according to the manufacturer's instructions. The inserts were sequenced from both directions using vector-specific primers, with an automated DNA sequencer. Each experiment was confirmed at least twice and the results were shown to be consistent.

## RESULTS

### Serial analysis of gene experssion and VNB for kallikrein expression in breast cancer

As shown in Table 3

**Table 3:** *In silico* analysis of kallikrein gene expression in normal and breast cancer tissues and cell lines screened by SAGE database

**Kallikrein**	**Library**	**Positivity (%)**	**Average density^a^**
*KLK3*	Normal breast	1/8 (13)	26
	Breast cancer	0/24 (0)	0
*KLK5*	Normal breast	5/8 (62)	319
	Breast cancer	1/24 (4)	34
*KLK6*	Normal breast	3/8 (37)	270
	Breast cancer	2/24 (8)	15
*KLK8*	Normal breast	2/8 (25)	27
	Breast cancer	0/24 (0)	0
*KLK10*	Normal breast	4/8 (50)	85
	Breast cancer	0/24 (0)	0
*KLK11*	Normal breast	3/8 (37)	40
	Breast cancer	1/24 (4)	28
*KLK12*	Normal breast	1/8 (13)	15
	Breast cancer	0/24 (0)	0

a Tags per million (tpm).

, seven kallikreins were found to be downregulated in breast cancer compared to normal breast tissues and cell lines. Probing all breast libraries from different sources (cancerous and normal tissues and cell lines) with *KLK5*-specific probes revealed that while *KLK5* was detectable in five out of eight normal breast libraries with moderate-to-high density (average expression of 319 tpm), mRNA tags were detectable in only one out of 24 cancer libraries. This library is a ‘carcinoma *in situ*’ and showed 34 tpm expression level. *KLK6*-specifc tags were also detectable in three out of eight normal libraries with high density (average 270 tpm) and in only two cancer libraries (out of 24) with low average expression level (15 tpm). *KLK8* showed a generally lower expression in normal breast (average 27 tpm). However, expression was undetectable in the 24 cancer libraries screened. Moderate *KLK10* expression levels (average 85 tpm) were detected in four out of eight normal breast tissues, but expression was undetectable in all cancer libraries tested. Slightly higher expression levels were detected for *KLK11* and *KLK12* in normal breast compared to cancer, although not statistically conclusive (Table 3). It is worth mentioning, however, that the only cancer library where *KLK11* was detected was described in the database as ‘Low grade malignancy, with some *in situ* component’. The results for *KLK3* were inconclusive, as low expression was detected in only one normal library, but not in cancer libraries (Table 3). A low number of *KLK1* and *KLK13* mRNA messages were detectable in the MCF7 breast cancer cell line 3 h after oestradiol stimulation (16 tpm for each gene), but not in unstimulated cells (data not shown). No significant change of expression levels was detected in the ZR-75 breast cancer cell line when stimulated by oestrogen (data not shown).

### Analysis of kallikrein gene expression utilising EST databases

Our EST library screening results (Table 4

**Table 4:** *In silico* analysis of kallikrein gene expression in normal and cancerous breast tissues and cell lines based on the EST databases

**Kallikrein**	**Library type**	**Positivity**	**Number of clones**
*KLK3*	Normal breast	0/7	0
	Breast cancer	1/14	2
*KLK5*	Normal breast	3/8	25
	Breast cancer	1/14	1
*KLK6*	Normal breast	5/8	32
	Breast cancer	1/14	1
*KLK8*	Normal breast	2/8	14
	Breast cancer	1/14	9
*KLK10*	Normal breast	5/8	25
	Breast cancer	1/14	1
*KLK11*	Normal breast	2/8	10
	Breast cancer	0/14	0
*KLK12*	Normal breast	1/8	2
	Breast cancer	0/14	0

) were consistent with SAGE expression and VNB data. While very few EST clones were retrieved from the 14 EST cancer libraries (nine for *KLK8*, two for *KLK3*, and one for each of the rest), EST clones were much more detectable in normal libraries. In all, 25 *KLK5* EST clones were detected in three out of eight normal breast libraries. *KLK6* showed higher expression levels; 32 EST clones were found in five out of eight libraries screened. Totally, 25 *KLK10* EST clones were detected in five out of eight normal libraries, and 14 clones were positive for *KLK8* in two out of eight normal libraries. Two EST clones for *KLK3* were detected in breast cancer. It should be emphasised, however, that EST figures are more useful as ‘qualitative’ data. Quantitative EST results are only approximate and cannot be relied upon, due to the fact that some EST libraries are normalised. No other kallikreins were found to be differentially expressed in breast cancer using SAGE or EST databases.

### X-profiler and DDD analysis of kallikrein gene expression in the breast

Table 5

**Table 5:** X-Profiler analysis of kallikrein gene expression in normal and cancerous breast tissues and cell lines

	**Gene expression counts**		
**Kallikrein**	**Cancer^a^**	**Normal^b^**	**Difference factor^c^**
*KLK5*	2	7	0.797
*KLK6*	2	6	0.716
*KLK10*	0	6	0.877
*KLK12*	2	4	0.719

a A pool of 22 breast cancer SAGE libraries.

b A pool of seven normal breast SAGE libraries.

c A factor of one represents the highest statistical significance.

shows the analysis of kallikrein gene expression in normal and cancerous breast tissues utilising the ‘X-profiler’ analysis tool. A pool of eight normal breast tissues and cell lines was compared to a second pool of 24 breast cancer tissues and cell lines (all non-normalised). Our results clearly indicate significant differences in the expression levels of four kallikreins in breast cancer compared to normal. *KLK5*, *6*, *10* and *12* were all downregulated in cancer compared to normal. The most significant result was obtained for *KLK10* (difference factor of 0.877).

No statistically significant differences were detected by the DDD, except for *KLK3*, where a 10-fold increase was found in cancer when comparing the pool of normal and breast cancer libraries.

### Evaluation of the *in silico* analysis

We verified the reliability of the *in silico* analysis with previously published reports on kallikrein expression in normal tissues (Yoshida *et al*, 1998; Yousef and Diamandis, 2000,^2003^; Yousef *et al*, 2001,2000b,2000d). These results were in general agreement with PCR and Northern blotting data (Gan *et al*, 2000; Clements *et al*, 2001; Yousef and Diamandis, 2001). For example, our SAGE and EST data indicate that *KLK2-4* are highly expressed in the prostate, while *KLK5* is highly expressed in normal breast tissue and skin, in agreement with published experimental reports (Brattsand and Egelrud, 1999; Yousef and Diamandis, 1999). In addition, we found that those kallikreins that show differential expression in breast cancer have similar levels of expression in normal and cancer tissues in other malignancies (data not shown), further verifying the specificity of our results.

### RT – PCR analyses in normal and cancerous breast tissues

**Figure 1:**
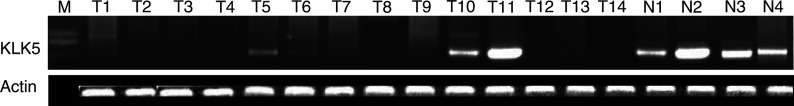
Expression of the *KLK5* gene in breast tumour (T1–T14) and normal (N1–N4) tissues. While high-expression levels were detectable in all normal tissues, the gene was expressed in only three out of 14 cancerous tissues. Actin, a house-keeping gene, was used as a control. M, molecular weight marker.

**Figure 2:**
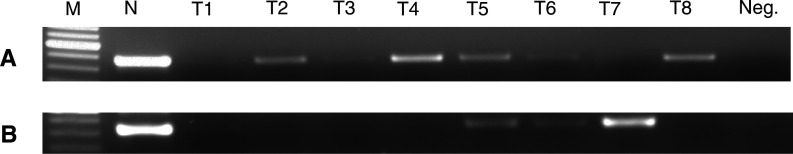
Representative gels showing differential expression of: (**A**) *KLK8*, (**B**) *KLK10*, in normal (N) and tumour (T) breast tissues. For discussion, see text. M, molecular weight marker.

**Figure 3:**
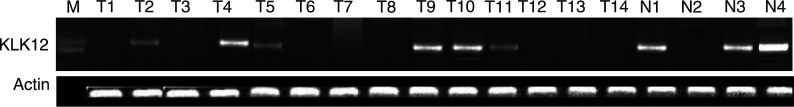
Expression of the *KLK12* gene in breast tumour (T1–T14) and normal (N1–N4) tissues. Actin, a house-keeping gene, was used as a control. M, molecular weight marker.

The expression patterns of two kallikreins were further verified for *KLK5*, *6*, *8*, *10*, *12* and *15* by RT–PCR analysis in histologically confirmed normal (*N*=4) and cancerous (*N*=14) breast tissues. As shown in Figure 1

, while *KLK5* was expressed at high levels in all four normal breast tissues, it was only detectable in three out of 14 cancerous breast tissues examined. *KLK6* was strongly positive in normal breast tissue, but was not expressed in nine out of 14 tumour tissues, lower than normal in three and compared to normal in two (data not shown). *KLK8* also showed a strong band in normal tissues, compared to undetectable expression in five tumour tissues, lower than normal in seven tumours and compared to or more than normal in two (Figure 2A

). *KLK10* showed no expression in eight tumours, lower than normal in five and compared to normal in one tumour (Figure 2). Figure 3

shows that while *KLK12* was detectable in three out of four normal breast tissues, it was expressed at relatively lower levels in six out of 14 cancers. We also analysed *KLK15* expression (as a negative control with no expression difference by *in silico* analysis), and found no difference in expression between normal and breast tumour tissues (data not shown).

## DISCUSSION

Our results indicate that at least four kallikrein genes are downregulated in breast cancer. These data are consistent with earlier reports indicating differential expression of kallikreins in breast cancer (Diamandis and Yousef, 2001; Diamandis and Yousef, 2002; Yousef and Diamandis, 2002a). The *KLK10* gene (previously known as normal epithelial cell-specific gene 1, NES1) was originally cloned by subtractive hybridisation based on its downregulation during oncogenic transformation of human mammary epithelial cells (Liu *et al*, 1996). More recently, it was suggested to have tumour suppressor activity (Goyal *et al*, 1998). Using a highly sensitive and specific immunoassay, we found hK10 expression in normal breast and in milk of lactating women (Luo *et al*, 2001b). hK10 was also found to be a potential marker for other malignancies, like testicular and ovarian cancers (Luo *et al*, 2001a,2001c,^2003^). Recently, Band and colleagues have shown, using *in situ* hybridisation, lower levels of *KLK10* expression in breast cancer cells when compared to normal cells (Dhar *et al*, 2001).

Our results with different tools (SAGE, EST and X-profiler) were consistent for *KLK5*, *6* and *10*. The results for *KLK3*, however, were not clear cut. Whereas the SAGE data showed a slight downregulation in cancer compared to normal, the EST and DDD results indicate an overexpression in breast cancer. Neither of the above results was robust enough to draw a conclusion. The SAGE data were obtained from only one library and the level of expression was ‘low’. The EST data were also only two clones from one library. In addition, some of the libraries used for DDD were normalised or subtracted, thus reducing the quantitative meaning of the results. This apparent discrepancy might be due to the overall low expression levels of *KLK3* in the breast, leading to undetectable transcripts in many libraries. Earlier, it was reported that *KLK3* (PSA) is not prostate specific and that it is also expressed in many other tissues including the female breast, albeit at much lower concentrations (Black and Diamandis, 2000). The potential prognostic importance of hK3 expression in breast cancer has also been reported (Yu *et al*, 1995).

Significant differences in expression levels were obtained for *KLK12*, especially when comparing the normal and cancer pools (Table 5). These results follow the general pattern of downregulation of kallikreins in breast cancer. Our previous results indicate moderate *KLK11* mRNA expression in the normal mammary gland (Yousef *et al*, 2000c). We have also previously shown that *KLK5* mRNA is highly expressed in normal breast tissue (Yousef and Diamandis, 1999) and found potential prognostic significance of *KLK5* expression in breast cancer (Yousef *et al*, 2002,^2003^).

The *KLK6* gene (previously known as zyme, protease M, neurosin, PRSS6) was originally cloned based on its lower expression in metastatic breast cancer compared to primary cancer and normal tissue (Anisowicz *et al*, 1996). In this study, the mRNA was detectable in one out of two normal cell lines, two out of three primary breast cancer and one out of nine metastatic breast cancer cell lines. Considering the small sample number in the previous study, it was difficult to draw a definite conclusion about the expression of this gene in different phases of cancer.

Nacht *et al* have recently used a novel approach of combining SAGE analysis with array technology to find genes differentially expressed in breast cancer (Nacht *et al*, 1999). Comparing one normal cell line *vs* two primary tumour cell lines, they identified *KLK6* as one of the differentially expressed genes between normal and primary breast cancer (a 2.4-fold increase in cancer). It is possible that these results reflect an individualised behaviour of certain cancer cell line. Our results have the advantage of comparing pools of normal and cancerous tissues, rather than individual cell lines. In a more recent analysis by Seth *et al*, kallikreins 5 and 6 were identified among 35 transcripts that were most abundantly expressed in oestrogen receptor (ER)-positive normal human breast tissue (Seth *et al*, 2002). Interestingly, comparing their constructed SAGE library tags with other normal, *in situ*, invasive and metastatic carcinoma tags from different SAGE libraries, their data indicate that these two kallikreins are exclusively expressed in normal breast tissues (Seth *et al*, 2002).

The hK6 protein was recently shown to be a potential marker for ovarian cancer (Diamandis *et al*, 2003). Also, in a recent analysis, the median serum concentration of hK6 was found to be higher in normal females (median expression of 7.0 *μ*gl^−1^) compared to breast cancer patients (median expression of 4.3 *μ*gl^−1^) (Diamandis *et al*, 2000a). In addition, the hK6 protein was found at high concentrations in breast tissue, milk of lactating women, nipple aspirate fluid and breast cyst fluid, with lower levels detectable in breast tumour cytosols (Diamandis *et al*, 2000b).

A noteworthy observation is the presence of a group of closely localised kallikreins (*KLK5*, *6*, *8*, *10*, *11* and *14*) with a similar pattern of differential expression in breast cancer. This points to the possibility of the existence of a locus control region or another common regulatory mechanism that controls the parallel expression of these kallikreins.

Other kallikrein genes, for example, *KLK13* and *KLK14*, were also previously reported to be downregulated in breast cancer (Yousef *et al*, 2000a,2000b,^2001^). Our analyses did not show any detectable expression of these genes by either SAGE or EST methods. This is likely due to the fact that these genes are expressed in the breast at very low levels that are not detectable, except by sensitive techniques like RT – PCR.

It should be emphasised that although *in silico* analysis is a useful research method, the results should be verified by more than one analytical tool, and further experimentally confirmed. Possible sources of bias include sequence errors, presence of specific sequence mutations associated with certain malignancies, unequal presentation of different physiological or pathological libraries and the expression of splice variants in certain malignancies.

Lercher *et al*, during global analysis of over 11 000 genes in 14 different tissues, have set a cut-off value for gene expression as being low (⩽37 tpm) and intermediate or high (⩾134 tpm) (Lercher *et al*, 2002). If this cut-off is to be applied to our data, we can conclude that while kallikrein gene expression is absent or low in breast cancer, medium-to-high levels of expression are found in the normal breast.

Several hypotheses can be proposed regarding the possible mechanism(s) by which kallikreins may be involved in cancer. *KLK3* was previously shown to have an antiangiogenic and a tumour suppressor effect on the growth of some breast cancer cell lines (Lai *et al*, 1996; Fortier *et al*, 1999). Breast cancer is a ‘hormonal’ malignancy. Kallikreins, being under steroid hormone regulation, may represent downstream targets by which hormones influence the initiation or progression of cancer. Another possible mechanism for the involvement of kallikreins in malignancy is the activation of proteinase-activated receptors (PAR). The activation of these receptors elicits different responses in several tissues.

It is important to mention that mRNA levels might not be necessarily be associated with a decrease in the serum or tissue levels of these proteins. As is the case with hK3, the serum rise might be due to ‘leakage’ from malignant cells due to architectural and angiogenic changes. We have recently shown elevation of hK5 levels in the serum of some patients with breast cancer (Yousef *et al*, 2003).

In conclusion, we provide strong evidence suggesting that at least four kallikreins are downregulated in breast cancer. These data were confirmed from several databases and by experimental analysis with RT – PCR. It will be useful to examine these kallikreins as biomarkers for diagnosis, prognosis and treatment decisions in breast cancer.
